# Quality variations of leachate resulting from cigarette filter recycling as a challenge for its management

**DOI:** 10.1038/s41598-024-51530-9

**Published:** 2024-01-10

**Authors:** Amin Hossaini Motlagh, Navid Alinejad, Farogh Kazembeigi, Javad Torkashvand, Hamid Reza Tashauoei, Mehdi Fattahi

**Affiliations:** 1 Department of Environmental Health Engineering, Faculty of Health, Yasuj University of Medical Sciences, Yasuj, Iran; 2 Department of Public Health, Fasa University of Medical Sciences, Fasa, Iran; 3 Student Research Committee, Ilam University of Medical Sciences, Ilam, Iran; 4 Department of Environmental Health Engineering, School of Health, Ilam University of Medical Sciences, Ilam, Iran; 5 Department of Environmental Health Engineering, School of Public Health, Iran University of Medical Sciences, Tehran, Iran; 6 Department of Environmental Health Engineering, Faculty of Public Health and Biomedical Engineering, Tehran Medical Sciences, Islamic Azad University, Tehran, Iran; 7 Institute of Research and Development, Duy Tan University, Da Nang, Vietnam; 8 School of Engineering & Technology, Duy Tan University, Da Nang, Vietnam

**Keywords:** Environmental sciences, Chemistry

## Abstract

Recycling is known as a solution for cigarette filter management, but this may cause the release of trapped pollutants in it. Cigarette smoke toxins and chemicals that trapped in the cigarette filter can accumulate in the recycling leachate. In this study, littered cigarette filters and freshly smoked cigarette filters were recycled and the resulting leachate was analyzed. The results showed that the minimum and maximum Chemical Oxygen Demand (COD) of the studied leachates were 2100 mg/L and 11,300 mg/L, respectively. The maximum temporal variation in the studied leachate quality was 74.28%, but the maximum spatial variation was 314.2%. COD in the freshly smoked sample was 2600–9200 mg/L more than the littered samples. The average concentration of chromium, lead, nickel, and cadmium in littered samples was 0.023, 0.024, 0.045, and 0.019 mg/L, respectively. Environmental conditions such as humidity, the efficiency of the urban cleaning system in reducing the resistance of littered filters, the difference in the quality of the filter and tobacco, and the difference in smoking behaviors were effective in this variation. Reducing the toxicity of cigarette smoke and improving the efficiency of the urban cleaning system can lead to the same quality, but leachate treatment is necessary to reduce the environmental risk.

## Introduction

Tobacco use, as one of the common phenomena in societies, has become one of the preventable causes of death and disease in humans, so that about six million deaths worldwide are attributed to tobacco use every year^1^. Filtered cigarettes are the dominant form of tobacco use in the world, and although this issue exists in all countries, the smoking rate is varying^1,2^. Some reports have stated that the production of filtered cigarettes in the world is 5.5 billion per year^3^. This high consumption of filtered cigarettes in the world not only has health consequences for smokers and people exposed to secondhand smoke, but it also has a serious environmental consequence, which is the cigarette butt^4^. Cigarette butt is the solid waste resulting from smoking, which includes remained tobacco and cigarette filter that its chemical characteristics and physical structure has changed due to the smoking process^5^.

The need to protect the health of smokers against the pollutants of cigarette smoke led to the production of filters and adding them to cigarettes^6^. In this way, the cigarette filter was designed to trap cigarette smoke pollutants, which reduced the health consequences of smoking, but led to the appearance of cigarette butt as an environmental consequence of smoking^3^. The trapping of thousands of chemicals and toxins, hundreds of which are carcinogenic, makes cigarette butt known as hazardous waste with various environmental and health effects^7^. Due to the variety of pollutants trapped in the filter, discarded cigarette butts known as an important source of heavy metal, Polycyclic Aromatic Hydrocarbons (PAHs), and BTEX (benzene, toluene, ethylbenzene and xylene) in urban environment^8,9^. Even the pollution of the coastal areas due to the leakage of various pollutants from littered cigarette butts and its environmental consequences on the marine ecosystem are serious concerns^10–12^. The management of this hazardous waste faces serious challenges due to its characteristics such as littering in urban and public environments, durability, containing many chemicals, and the lack of a safe method for final disposal^13^. Serious concerns regarding the release of polluting gases into the atmosphere due to cigarette filters’ incineration, as well as the leakage of various pollutants such as heavy metals from landfill leachate into the soil and water sources, have caused the use of conventional solid waste disposal methods such as incinerators and landfills for cigarette filters not recommended^14^. In this situation, focusing on recycling as the goal of cigarette filter management is suggested as a suitable approach and solution^3^.

Although cigarette filters are an important threat to the environment^7^, but the results of recent studies have shown that it can be used for recycling and producing useful products^15^. Cigarette filters can be recycled in three general categories. In the simplest category, the cigarette filter is used without separating its components. In this category, which is called a process-free, a certain number of cigarette filters are mixed with raw materials^15^. For example, the production of bricks and asphalt is included in this category^16,17^. In the other two categories, which are called recycling that requires processing, the recycle goal components separate from the cigarette filter. These components include two groups, which are cellulose acetate used in the cigarette filter^18^ and chemicals trapped in the cigarette filter^18^. Accordingly, the use of filters and trapped chemicals can be included in the triple category of cigarette filter recycling.

Cigarette filter can be recycled into activated carbon, biofilm barrier, porous sound absorber, and super hydrophobic fibers^19–22^, but strong leachate as a recycling by-product is considered a serious challenge. Leakage of chemical compounds trapped in cigarette filters is one of the challenges of recycling this hazardous waste. This problem is especially greater in filter recycling methods because the pollutant removal processes from smoked filters by washing will lead to the production of strong leachate^23,24^. Also, due to the influence of smoking behavioral factors, climatic conditions, and the quality of cleaning services, the concentration of pollutants in the filter is not the same. These conditions cause the quality of leachate resulting from the recycling of this hazardous waste to be different. So far, few studies have been conducted on the environmental consequences of cigarette filter recycling, and there is no information on the quality of the leachate resulting from their recycling. Considering that the production of leachate is inevitable in the cigarette filters recycling, predicting its management steps is effective in reducing the subsequent environmental consequences. One of the requirements for management of leachate resulting cigarette filter recycling is to know the quality of this type of leachate and the factors affecting it. Therefore, variation in the quality of leachate is the most important difficulties and challenges in cigarette filter recycling, which will cause a shock in its treatment process. Predicting the ratio of pollutant concentration changes in leachate resulting from filter recycling is necessity to control the treatment process and overcome the shock caused by leachate quality variation. The aim of this study was to investigate the quality of leachate resulting from the recycling of different cigarette filter samples based on temporal and spatial variations. Knowing the quality of leachate resulting from filter recycling and the factors affecting it, which is presented for the first time in this study, can be useful in completing the management steps of this hazardous waste and controlling its environmental consequences.

## Method

### Cigarette filter samples

This study was done by processing eighteen samples from six categories of samples of cigarette filters and investigation the quality of the resulting leachate. The processed cigarette filters included two main groups of Freshly Smoked Cigarette Filters (FSCF) and Littered Cigarette Filters (LCF), six categories of studied samples consist one sample of FSCF and five samples of LCF. FSCF refers to cigarette filters prepared from smoking filtered cigarettes in a laboratory and is an example of disposed cigarette filters by smokers in the waste containers or trash bins. LCF refers to cigarette filters that littered by smokers then collected from the urban environment or public areas by urban cleaning systems. This classification was aimed to investigating the difference in the quality of leachate from recycling caused by the difference in the management of cigarette filters in the stages of storage and collection. LCF samples were collected from the streets, parks, and alleys of Tehran, Iran. The FSCF sample was prepared from most popular brands in Iran. LCF samples were collected in three land-uses including commercial (LCF1), residential (LCF2), and recreational (LCF3). These samples were collected to investigate the changes in leachate quality due to the effect of land-use on the durability of cigarette fillers in the urban environment. In each land-use, samples were collected from three different locations. To investigate the effect of temporal variation, sampling of commercial land-use was done at three different times (LCF1,T1-LCF1,T3). In total, fifteen LCF samples and three FSCF samples (18 samples) were studied. Finally, the average results were calculated for each land-use. The samples were collected using the field observation protocol in LCF studies^25^. LCF samples were collected in the evening hours from three locations in different land-use on working days^26^. First, LCF samples were collected in one day from three locations in different land-uses. Then, for investigate the effect of temporal variation, two more samples were collected with an interval of one month. The preparation of FSCF samples and collected LCF samples included three stages of remained tobacco separation, plug wrap and wrapping paper separation, and filter cutting.

### Leachate samples

**Figure 1:**
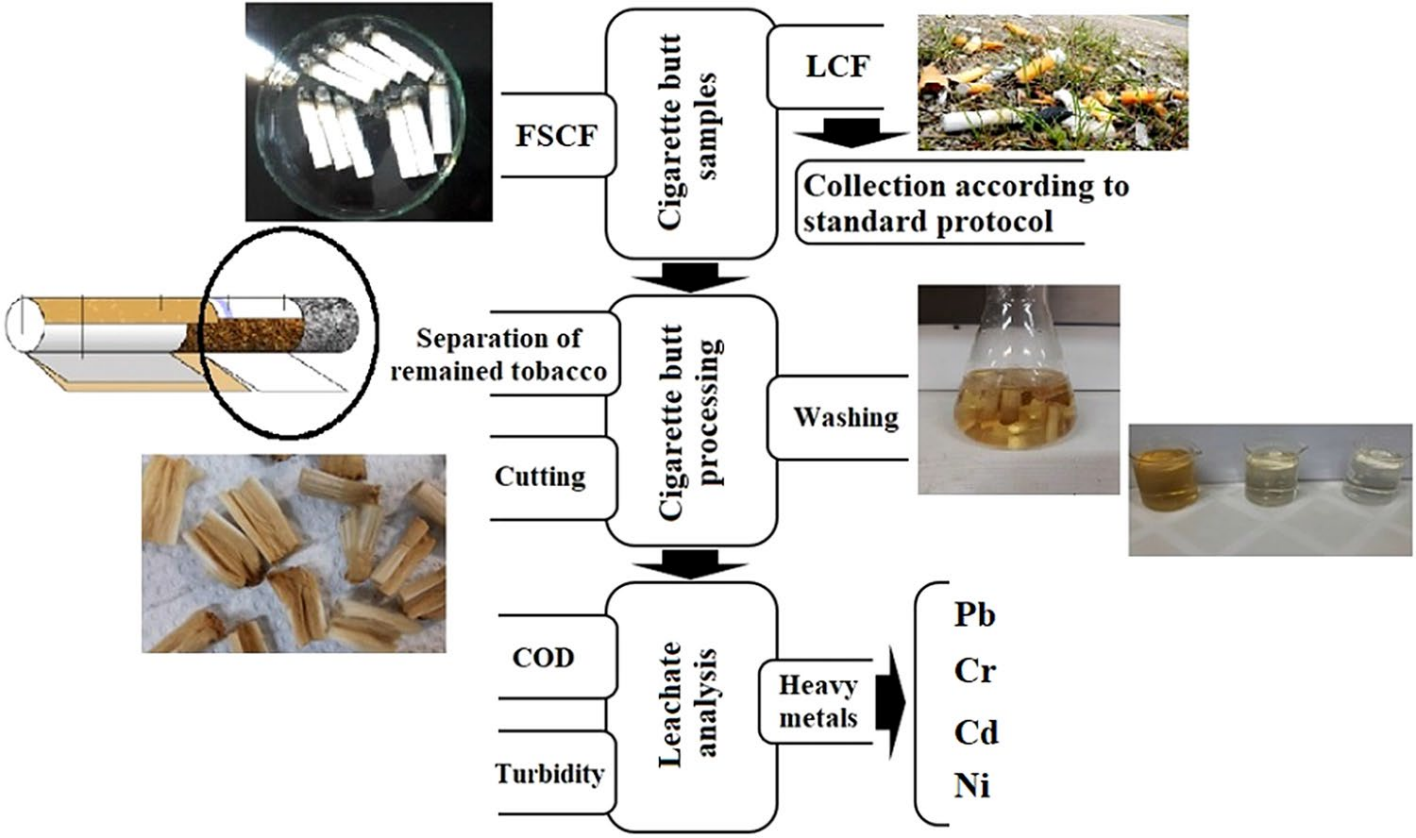
Study steps include sampling, processing, and leachate analysis.

Leachate samples were prepared by the processing shown in Fig. 1 for each of the cigarette filter samples. For this purpose, the common method of recycling the filter into activated carbon, biofilm barrier, porous sound absorber, super hydrophobic fibers and the like was used^24^. For this purpose, after transferring the cigarette filter to the laboratory in the first step, the remained tobacco was removed manually and the filters were cut as shown in Fig. 1. The washing steps leading to the production of leachate based on previous studies in cigarette filter recycling^18,22,23,27^ included immersion and mixing in water for twenty minutes for three times. Then the filters were immersed in 96% ethanol for 20 min for two times^24^. The mixture of the effluents from washing the cigarette filters with the effluents from washing the containers used for immersion of cigarette filters in ethanol was studied as leachate samples.

### Sample analysis

In this study, the chemical oxygen demand (COD) of cigarette filter processing leachate samples was performed based on closed reflux method (5220 standard method). In this method, a special vial with three different concentration ranges is used, which are: 0–150 mg/L, 0–1500 mg/L, and 0–15,000 mg/L. After passing through 0.45 µm membrane filters, 2 cc of the sample was added to a dedicated vial and placed in a preheated digester (150 °C) for 2 h. Then, the sample was placed at room temperature (25 °C). The chemical oxygen requirement of the sample was read using a MN DR-6000 Spectrophotometer and a wavelength of 600 nm.

To analysis of heavy metals, standard method No. 3111 was used. In order to determine heavy metals, after preparing the samples, first, using metal salts, standard solutions of 1000 mg/L were prepared. Based on the type of metals, the solutions used for the calibration of the atomic absorption were prepared from a stock solution of 50 mg/L with concentrations of 0.1, 1, 3, 5, and 10 mg/L. Then, the calibration curve (concentration in terms of absorbance) was drawn using these solutions, and finally, the samples were analysis by the atomic absorption (Varian 220 Spectr AA).

## Results and discussion

**Figure 2:**
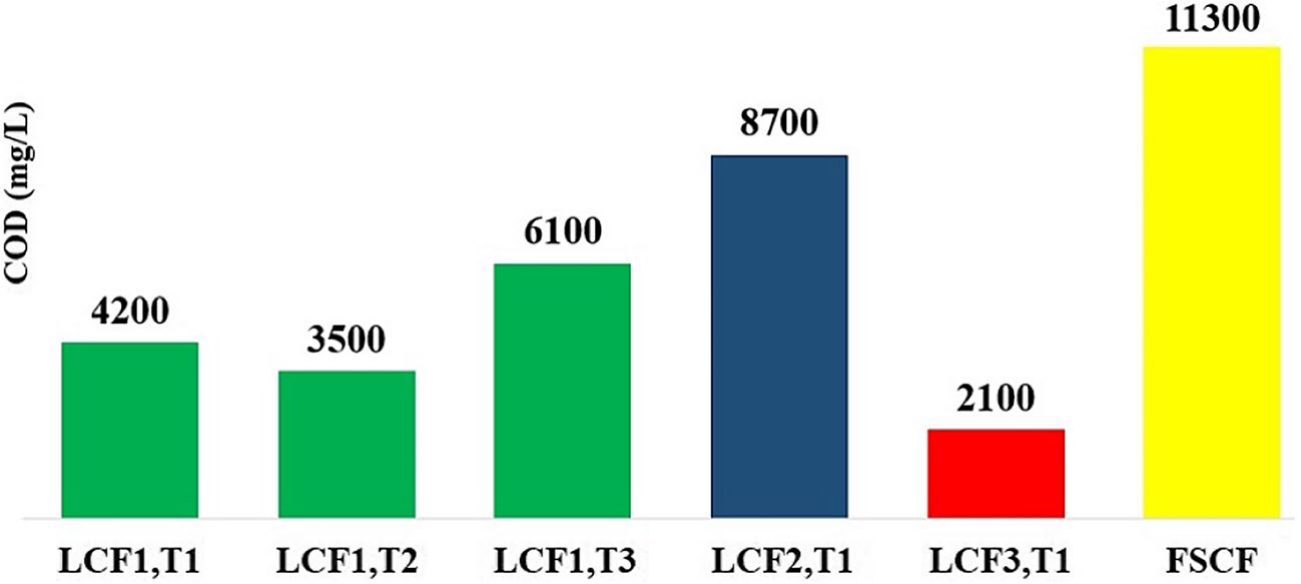
COD of leachate resulting from the processing of different samples of cigarette filters (mg/L). T1, T2, and T3 represent the first sampling at time 1, the second sampling at time 2, and the third sampling at time 3, respectively.

The results of COD analysis of six leachate resulting from the processing of six cigarette filter samples are shown in Fig. 2. The results showed that COD of leachate resulting from cigarette filter recycling had spatial and temporal variation. COD of leachates resulting from LCF collected from location 1 in a two-month interval was in the range of 3500 to 6100 mg/L. In this way, it was observed that LCF processing at different times led to different quality of leachate. Also, the difference in COD of leachate resulting from LCF processing collected from three different locations was also in the range of 4200 to 11,300 mg/L which indicated the difference of quality of leachate resulting from LCF processing that littered in different locations. In addition, a significant difference was observed in the COD of leachate resulting from LCF recycling and leachate resulting from FSCF recycling. As shown in Fig. 2, the difference in COD of FSCF leachate with the COD of weakest and strongest LCF leachate was 438% and 30%, respectively.

Although the COD of leachates from the recycling of different cigarette filter samples had a significant difference, but an insignificant difference was observed in the turbidity. As shown in Table 1, the results showed that the turbidity of FSCF leachate was different from the average turbidity of LCF samples. Therefore, temporal and spatial variation in leachate turbidity caused by cigarette filter recycling were very minor. However, as shown in Table 1, the spatial and temporal variation in the heavy metals concentration of recycling leachates did not have the same trend. For example, the difference in lead concentration in FSCF leachate was greater than the average lead concentration in LCF leachate by more than 91.6%. This difference in the case of chromium, cadmium, and nickel was 33, 12.2, and 25%, respectively. However, it was observed that the concentration of all studied metals in FSCF leachate was higher than LCF leachate.

**Table 1:** Concentration of heavy metals and turbidity in leachate resulting from the processing of cigarette filter samples.

	Turbidity (NTU)	Lead (mg/L)	Chromium (mg/L)	Cadmium (mg/L)	Nickel (mg/L)
FSCF	119	0.046	0.031	0.022	0.057
LCF1	113	0.023	0.027	0.020	0.047
LCF2	124	0.019	0.022	0.017	0.041
LCF3	97	0.031	0.021	0.022	0.049

As the results showed, despite the fact that the same processing method was used for all the cigarette filter samples, the quality of leachate resulting from the cigarette filter processing was significantly vary in different samples. These conditions can be a concern in the cigarette filters recycling because the leachate resulting from recycling of cigarette filters will have different quality in each period or day, which will make the operating difficult. Therefore, knowing the reasons for the temporal and spatial quality differences of recycling leachate is necessary for the design of the treatment system because one of the challenges of cigarette filters recycling is the production of strong leachate^24^. In order to find the reason for the quality difference of recycling leachate, it is necessary to pay attention to four categories of affecting factors, including filter pollution, climatic conditions, cigarette filter management, and the smoking behavior.

The difference in pollutant concentration in different samples of cigarette filters is one of the reasons for the qualitative difference in recycling leachate. The concentration of pollutants such as heavy metals in cigarette filters of different brands was reported vary^28^. This difference is due to two main reasons, which are the quality of the filter in trapping cigarette smoke pollutants and the difference in pollutants in the cigarette smoke, which is caused by the effect of the origins of pollution^24^. The cigarette filter is made of cellulose acetate fibers impregnated with plastic^15^, the difference in density and quality of the filter in different brands of cigarettes causes the difference performance in trapping cigarette smoke pollutants. Therefore, cigarette filters from smoking different brands have different concentrations of trapped pollutants, which will cause the variation in quality of recycling leachate. In addition, as shown in Table 2, the origins of cigarette smoke pollutant include four groups that are different in the production of cigarette brands. The origin of cigarette smoke pollutants includes the soil of tobacco cultivation, the nature of tobacco, pesticides used in tobacco cultivation, and additives to tobacco in the cigarette production processes in the factory^29,30^. The production of different types of cigarettes from different tobaccos that are produced in different regions and under different cultivation conditions, as well as the use of different additives in different cigarette factories, causes the variation in potential for the presence of pollutants in the cigarette smoke of different brands. Therefore, regardless of the filter's ability to trap pollutants, cigarette smoke from different brands has different pollutants that affect the quality of cigarette filter recycling leachate.

**Table 2:** Origins of cigarette smoke pollutant^19,23–25^.

Row	Categories	Example
1	The nature of tobacco	Nicotine
2	Agricultural soil	Heavy metals
3	Agricultural additive	Pesticide, herbicide, insecticide, fungicide
4	Cigarette additive	Brightening, solvent, plasticizer

**Figure 3:**
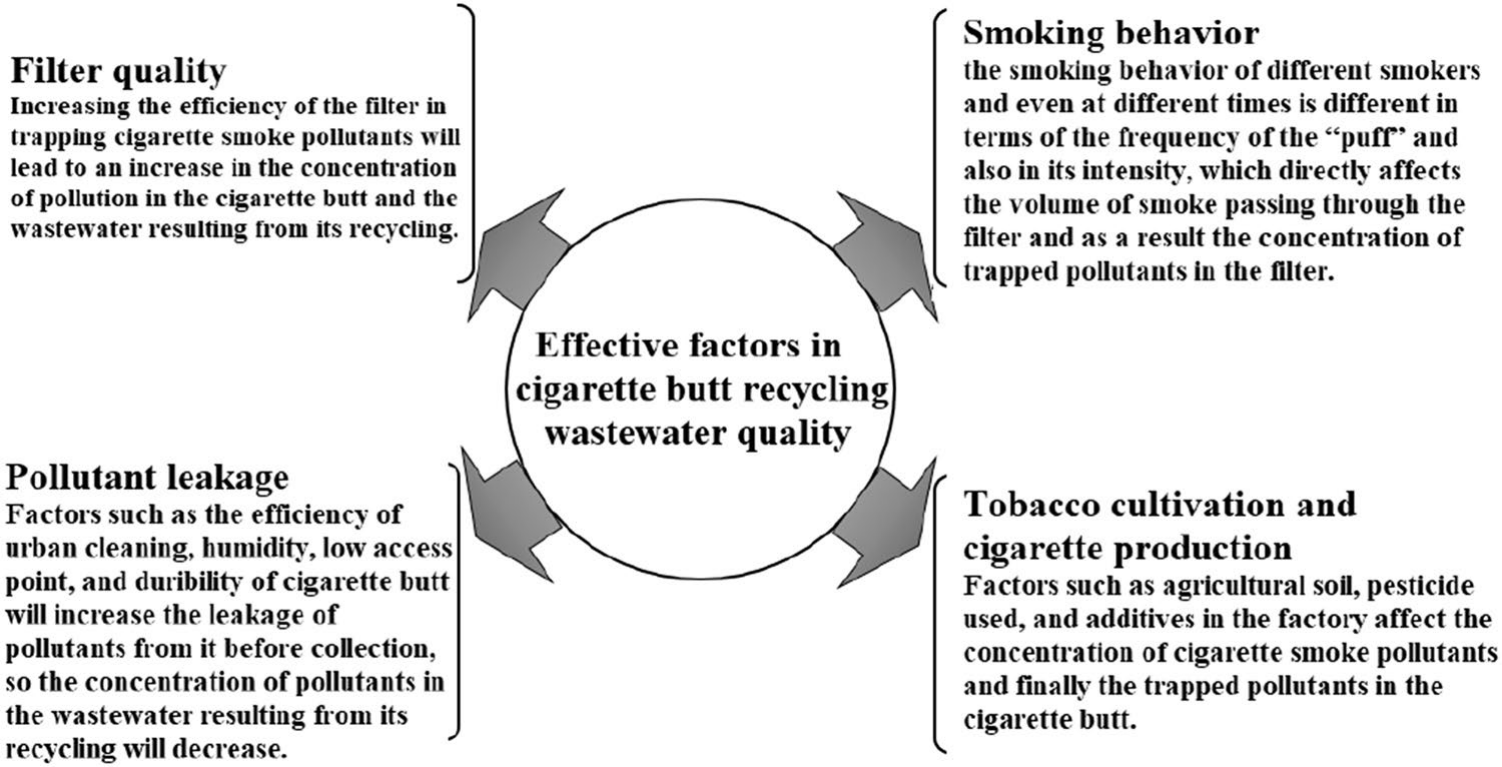
Affecting factors on the quality of studied leachate.

The amount of pollutant leakage from LCF before collection is one of the most important reasons for reducing the concentration of pollutants in leachate resulting from LCF recycling compared to FSCF. This phenomenon was reported in previous studies in the difference in the concentration of various types of pollutants in mainstream smoke and discarded cigarette butts^31^. The ratio of pollutant leakage from LCF to the environment is not the same. For example, it has been reported that the leakage of naphthalene, acenaphthylene, acenaphthene, and fluorene were significantly higher than other types of PAHs^32^. Climatic conditions such as humidity have an important effect on the leakage of trapped pollutants from littered cigarette filters^33,34^. For example, the leakage of nicotine from the cigarette filter is more during the rain, and also littering the cigarette filter into the river or the sea will lead to more PAHs leakage from it^35,36^. Therefore, the change of weather conditions, including precipitation, is more effective in reducing the trapped pollutants in cigarette filters due to leakage, which causes the change of recycling leachate quality. In other words, the greater amount of pollutant leakage from the littered cigarette filter to the environment before collection will lead to a greater reduction of the pollutant in the recycling leachate. As shown in Fig. 3, in addition to the quality of the filter and the effect of the origins of pollution, two other factors have an impact on the pollution concentration in cigarette filter and the quality of recycling leachate, which are the efficiency of the collection system and smoking behavior.

An effective factor in the quality of recycling leachate is the residence time of littered cigarette filters in the environment. As the residence time increases, the quantity of pollutant leakage from LCF will increase, and as a result, the pollutant concentration of recycling leachate will be lower. This point will be more important when we notice that the rate of leakage of all pollutants from the littered cigarette filter is not the same. The leakage of some pollutants from LCF reaches its peak after a few days, while some pollutants leak quickly after the cigarette filter littering^7,35^. Considering that the leakage of pollutants from LCF in the urban environment reaches its peak a few days after littering^37^, the lower durability of LCF due to the optimal efficiency of the urban cleaning system can reduce the leakage of pollutants into the environment and increase the concentration of pollutants in the leachate resulting from recycling. Therefore, effective factors in the collection efficiency of littered cigarette filters such as the frequency of urban cleaning, low access points such as three-pits, and the characteristics of the environment such as ratio of grassy area or asphalt quality^38^ indirectly affect the quality of recycling leachate. In addition, a very important factor in the amount of trapped pollutants in the cigarette filter and as a result the concentration of pollution in the cigarette filter is the smoking behavior^39,40^. In fact, the smoking behavior of different smokers and even at different times is different in terms of the frequency of the “puff” and also in its intensity, which directly affects the volume of smoke passing through the filter and as a result the concentration of trapped pollutants in the filter. Also, the ratio of remained tobacco on the filter is different in each smoking. So, considering that part of cigarette filter pollution depends on remained tobacco, the pollutant concentration in different cigarette filters will not be the same. Therefore, one reason for the difference in recycling leachate quality is the difference in pollutant concentration in it due to the different smoking behavior. Therefore, littering cigarette butts in the environment is not only a threat to the ecosystem due to the leakage of many compounds such as aromatic amines^41,42^, metals^8,10^, nicotine^12^, and PAH, but also led to the variation in the quality of by-products, which is a serious challenge in the next stages of cigarette butt management.

As the results showed, the quality of FSCF leachate was significantly different with the quality of LCF leachate. These results show the impact of cigarette filter management method as a hazardous waste in its subsequent environmental consequences^7,24^. According to the behavior of smokers in littering cigarette filters^28,43^, there are two general routes to manage this waste, which includes collecting littered cigarette filters and management relying on correct disposal of cigarette filters in trash bins. Considering the effect of climatic and environmental factors on the spatial and temporal variation in LCF leachate, the use of cigarette filter management model based on its correct disposal in the trash bins that leads to FSCF can reduce the subsequent problems of leachate treatment. Therefore, although the leakage from the littered cigarette filters will reduce the concentration of pollutants in the recycling leachate^24^, but the correct disposal of the cigarette filters will reduce the variation in the quality of recycling leachate^28^. Also, this management method will reduce the health and environmental consequences of pollutant leakage from the littered cigarette filters^33,35^. Reducing cigarette filter lettering by educating smokers and changing their behavior, installing equipment and bins for cigarette filter disposal in public areas, creating anti-lettering laws and tax, and the use of extended producer responsibility^40,44–46^ can be achieved.

This study had strengths and limitations. This study was the first comprehensive investigation of the quality of leachate resulting from cigarette filter recycling. The variation of leachate quality resulting from the recycling of different cigarette filter samples affected by the behavioral factors of smokers, climatic conditions, and the durability of the filter in the environment was investigated for the first time in this study. Although four heavy metals, turbidity, and COD were among the studied variables, other pollutants trapped in the filter such as PAHs, other metals, and nicotine^12^ can be studied in the future. This study comprehensively examined the quality of leachate, but the investigation of various treatment methods to choose the appropriate method for the treatment of leachate resulting from cigarette filter recycling was another limitation of this study.

## Conclusion

The quality of leachate resulting from cigarette filter recycling was studied. Two groups of cigarette filters, including *Freshly Smoked Cigarette Filter* and *Littered Cigarette Filter*, were processed based on the most common cigarette filter recycling methods. The results showed that recycling leachate had significant spatial and temporal variations in the investigated parameters, including COD, turbidity, and heavy metals such as chromium, cadmium, nickel, and lead. The maximum difference of COD, turbidity, nickel, lead, chromium, and cadmium in the studied samples was 438, 28, 25, 91, 33, and 12%, respectively. The total concentration of studied metals was 0.111 mg/L. The highest and lowest ratio was associated with lead (40.54%) and nickel (17.11%), respectively. Therefore, leachate quality variation is one of the challenges of cigarette filter recycling, especially in the operation of its treatment process. The variation in the quality of leachate is caused by the following:

1. The difference in the smoking behaviors that effective in the volume of passed smoke through the filter
2. The difference in filter quality is effective in the concentration of pollutants trapped in it
3. Land-use, which is effective in the durability of the littered cigarette filter in the environment and reducing its pollutants caused by leakage
4. Climatic conditions that effective in the quantity of pollutant leakage from the littered cigarette filter

Reducing the cigarette filter littering and increasing the proportion of freshly smoked cigarette filter will increase the pollutant concentration in recycling leachate, but it will reduce its quality variation and make it easier to operating treatment.

## Data Availability

The datasets generated and analyzed during the current study available from the corresponding author on reasonable request.
